# Curriculum Innovations: Creation of a Longitudinal, Neurology-Centered Pipeline Program to Motivate and Support Students From Racial/Ethnically Marginalized Groups

**DOI:** 10.1212/NE9.0000000000200007

**Published:** 2022-10-18

**Authors:** Shane Fuentes, Rachel M.E. Salas, Olivia Brumfield, Robert Thompson Stone

**Affiliations:** From the University of Rochester (S.F., O.B., R.T.S.), NY; and Johns Hopkins University (R.M.E.S.), Baltimore, MD.

## Abstract

**Introduction:**

Premedical students who identify from historically marginalized racial and ethnic backgrounds are more likely to lose interest in medicine than their White counterparts. Loss of interest has been attributed to a lack of exposure to the field and little mentorship.

**Objectives:**

The PreDoc Program was designed as a longitudinal experience to promote exposure to and interest in academic medicine, particularly through the lens of neurology for premedical students who identify from historically marginalized racial and ethnic backgrounds.

**Methods and Curriculum:**

The program included the following core components: (1) senior (faculty) mentor to facilitate direct contact with a physician, networking, and professional development coaching; (2) junior (medical student) mentor to provide near peer support and increased knowledge of the medical school application process; (3) large group meetings aimed at teaching professional development and working through clinical problem-based learning; (4) shadowing experiences aimed at increasing knowledge of patient care delivery and other academic roles; and (5) a clinically oriented project. After initial grant support to create the program, it has been maintained successfully with minimal funding through the Department of Neurology.

**Results and Assessment:**

The program recruited 29 student participants who completed at least 1 year of the program, 18 senior mentors, and 23 junior mentors over 4 academic years. The overall quality of the program was rated at 4.7 of 5 (median 5, range 2), with an upward trend seen over time. Over its first 2 years, the program facilitated the following estimated activities: 45 in-person senior mentor meetings, 27 in-person junior mentor meetings, 42 shadowing experiences, 60 large group meetings, and 360 email communications. Student-reported strengths included ease of shadowing, usefulness of problem-based learning cases, mentor relationships, and encouragement received. Areas for improvement included increasing the strength of junior mentor relationships and increased opportunities for socialization outside of the formal meetings.

**Discussion and Lessons Learned:**

It is feasible to create a successful, longitudinal, clinically focused undergraduate pipeline program for students who identify with historically minoritized racial and ethnic backgrounds with minimal funding centered in a Department of Neurology to help promote diversity within the field.

A disparity exists between racial and ethnically marginalized populations and their representation in medicine. As of 2018, 5% of physicians identified as Black or African American and 5.8% identified as Hispanic or Latinx, while 13.4% and 18.5% of the US population identified as Black or African American and Hispanic or Latinx, respectively.^1,2^ Despite efforts to overcome this disparity, the percentage of Black male physicians did not change between 1940 and 2018.^3^ In addition, the percentage of Black female physicians grew by only 2.7% in this time.^3^

These disparities in the healthcare workforce have also been observed in the field of neurology. From 2006 to 2017, 3.4% of neurology faculty assistant professors identified as Hispanic, Latino, or of Spanish origin and 2.5% identified as Black or African American. The percentages of individuals identifying with these backgrounds at higher levels of academic rank were even less.^4^ In addition to the lack of equity for Black and Latinx individuals with an interest in neurology, this disparity likely contributes to the underutilization of neurologic services and worse neurologic outcomes for patients who identify with similar backgrounds. From 2006 to 2013, 1.18% of the US population who identified as Hispanic and 2.06% of those who identified as Black visited a neurologist, compared with 3.26% of the White population.^5^ Distrust of a predominantly White physician workforce by Black and Latinx patients and the potential effect on health outcomes have been well documented.^6-9^ Black and Hispanic patients seen by physicians of a concordant race were more likely to rate care as excellent, including respect, explanations of medical problems, and listening.^10^ African-American patients being treated in a primarily African-American provider clinic were more likely to report trust, comfort, and the feeling of concern from the provider.^11^ Latinx patients have shown decreased satisfaction with their communication with White physicians.^12^

Many declared premedical students who identify with historically minoritized racial and ethnic backgrounds begin their undergraduate degree with intentions of becoming a physician. However, these students are less likely to remain premed than their White counterparts. Frequently cited reasons for students losing interest in medicine include limited knowledge of the field and minimal encouragement and mentorship. By contrast, engagement with physicians helps maintain interest.^13^ Generally, students accepted to medical school attribute their acceptance to guidance, information, and support from mentors and peers.^14^ Undergraduate students majoring in neuroscience reported that only 25%–30% had spoken to a neurologist about career experience and/or had the opportunity to shadow a neurologist/neuropsychologist. Furthermore, only 14% had the opportunity to interact with neurology patients. Whereas 96% of students who engaged in such interactions felt they were useful in characterizing the career of a neurologist. Those who were able to create these opportunities, most commonly did so using personal connections, which has the high potential to favor those in privileged positions over marginalized populations.^15^ Many short, research-focused programs (e.g., Summer internships) exist to increase readiness for the medical school application process, but there is a shortage of longitudinal clinically oriented programs directed toward students from racial or ethnically marginalized backgrounds.^16^

## Objectives

Drawing on the experience of the Johns Hopkins Medicine (JHM) Neurology Department, we created our own PreDoc Program at the University of Rochester. JHM's program successfully promoted undergraduate student interest and scholarly productivity in health care through longitudinal clinical, research, and educational exposure in academic medicine.^17^ We sought to design a longitudinal, 2-year, clinically focused experience for premedical students who identified with a historically marginalized racial or ethnic background. The program's goal was to promote exposure to and interest in academic medicine, particularly neurology, because the program was run through the Department of Neurology. In addition, given the limited sources of funding for pipeline programs, we sought to establish a model that would need minimal financial support to function effectively. The goals of the program were to inspire students to pursue careers in academic medicine and particularly neurology, address knowledge gaps about the medical school admission process, and provide students with meaningful clinical experiences and mentorship.

## Methods and Curriculum

Starting July 2018, undergraduate students at the University of Rochester and local Rochester area high school students were selected to participate in a 2-year longitudinal premedical mentorship program, titled the University of Rochester PreDoc program. Participants were required to self-identify with a racial or ethnic background historically minoritized in medicine, using the Association of American Medical Colleges definition of underrepresented in medicine: “Underrepresented in medicine means those racial and ethnic populations that are underrepresented in the medical profession relative to their numbers in the general population.”^18^ The program began with a 2-year pilot, which included the 2018–2020 academic years, and has continued annually since. The program runs throughout the academic year (September through April), allowing students to pursue alternative Summer experiences. Sharing of results from our program development and evaluation was deemed by the University of Rochester research subjects review board to not require oversight.

### Recruitment

During its first year, the program recruited 5 high school students in addition to 5 undergraduate students. There was a formal application, which circulated electronically to the University of Rochester Minority Association of Premedical Students group^19^ and the University of Rochester Science and Technology Entry Program (STEP) for high school students. The application included the following components: (1) personal statement, (2) unofficial transcript, (3) 1 letter of recommendation, (4) statement of interest and availability, and (5) demographic information. We considered a wide array of attributes during the selection process that attempted to weigh the relative level of privilege that students may have had already. For example, we favored first-generation college students and those who did not have the benefit of having a specific familial role model within medicine (learned through their personal statement). We examined grade point average (GPA) and valued those who showed academic success, but not necessarily scores equal to the average GPA for students admitted to medical schools. We also valued those with a lower than average GPA, but with a clear trajectory for improvement. We did not focus significantly on writing ability or access to previous medical experiences. During the first year, we received approximately 30 applications for 5 undergraduate spots and 5 high school students were chosen of 10 appropriately aged students in STEP. There was no interview process. After the first year, program content was deemed more suitable for undergraduate level learners, and only undergraduate students were recruited in the following years.

### Program Components

The major components of the program included senior (i.e., faculty) and junior (i.e., medical student) mentors, shadowing opportunities for both clinical experiences and other academic roles (e.g., conferences, teaching), large group meetings, and an individual clinical project (Table 1). Each participant was expected to meet with each of their mentors at least twice yearly. In addition, participants were expected to shadow their senior mentor initially and then with other physicians. At the University of Rochester, there is a short-term observational experience policy that allowed us to easily set up these experiences through a quick process involving an independent review and signing of materials by the student, and preceptor confirmation of student vaccination status. Through the shadowing and mentor experiences, students were expected to benefit from direct contact with a physician, increased knowledge about patient care delivery, advice about medical school preparation and applications, networking for research and volunteer opportunities, and the potential for letters of recommendation for Summer experiences and medical school applications. We did not specifically capture demographic information (e.g., self-identified race/ethnic background and sex) for our mentor groups and did not specifically attempt to recruit senior mentors who identified with a racial or ethnic background minoritized in medicine. We did attempt to recruit junior mentors who self-identified with similar race/ethnic backgrounds as our program students by indicating that desire in our recruitment messaging but did not require that the medical students provide us with their demographic information.

**Table 1:** PreDoc Program Components

**Program component**	**Description**
Senior mentor	Physician faculty at the University of Rochester who meets at least twice yearly with student, helps set up shadowing experiences, provides professional development guidance, and serves as a resource for professional letters of recommendation
Junior mentor	Medical student at the University of Rochester who meets 2–4 times yearly with student, provides professional development guidance and information about medical school applications.
Shadowing experiences	Students are provided 3–4 shadowing experiences per year with their senior mentor as well as other physicians at the University.
Large group meetings	Opportunities for the whole group to get together with program leadership, go over professional development topics, meet with panels of medical students and physicians, and participate in problem-based learning cases.
Individual clinical project	Individual project related to patient-care or a medical condition.

Students participated in 4–6 large group meetings per year to learn about and discuss important topics in medicine and professional development. Each meeting was 2.5 hours and began with a professional development talk or panel discussion (Table 2) followed by a medical problem-based learning case. The case topics rotated through Emergency Medicine, Internal Medicine, Neurology, Obstetrics and Gynecology, Pediatrics, Psychiatry, and Surgery. Large group meetings were expected to be in-person; however, the coronavirus disease 2019 (COVID-19) pandemic forced large group meetings between March 2020 and August 2021 to be virtual.

**Table 2:** Large Group Meeting Topics

Resilience and perseverance in the journey to medicine
Experience of a physician identifying with a racially or ethnically minoritized background
The power of (counter)-narratives: a focus on identity and implicit bias
How to be an effective mentee
Q&A panel with medical students who identify with a racially or ethnically minoritized background
Q&A panel with subspecialty physicians
Meeting with the dean of admissions at the University of Rochester
Meeting with the director of the office of diversity and inclusion
American Medical College Application Service overview

Throughout the 2-year program, students worked on an individual project of their choosing to present to their peers at the program graduation ceremony. Projects included medical research studies, patient informational pamphlets, and public health projects.

### Program Leadership

During the pilot, 1 leadership position existed: Program Director, who would oversee the program in its entirety. After the pilot, 2 additional leadership positions, Program Chief and Student Director, were created to spread the responsibilities among multiple individuals and provide students with leadership experience. Once the program was established, it is estimated that each leadership position has required approximately 20 hours per year of work in maintaining the program (not including the time allocation for individual student mentorship). The roles and responsibilities of all leadership positions are outlined in Table 3.

**Table 3:** PreDoc Program Leadership Roles and Responsibilities

**Role**	**Program Director: Physician20 h/y**	**Student Director: Medical Student20 h/y**	**Program Chief: Undergraduate student and graduate of the PreDoc program20 h/y**
Overall program administration	• Oversees program in its entirety • Manages program funding	• Serves as a liaison for the junior mentor/mentee relationship	• Notifies students of logistics, yearly schedule, and mentor-mentee pairings • Works with Alumni Chair to create yearly PreDoc Newsletter • Serves as Alumni Chair the following academic year • Annual website update
Recruitment	• Oversee student selection with other leaders • Recruits yearly cohort of faculty senior mentors and creates pairings	• Conducts detailed review of applications and helps select students with program leadership • Recruits yearly cohort of medical student junior mentors and creates pairings	• Conducts detailed review of applications and helps select students with program leadership • Communicates with, and conducts a teaching session for the MAPS program to increase interest in program
Coordinating, planning, and leading sessions	• Leads yearly senior mentor training session • Helps recruit guest speakers for large group meetings, attends most meetings, and facilitates problem-based learning cases	• Leads yearly junior mentor training session • Aids in coordinating, attends all large group sessions, and facilitates problem-based learning cases • Coleads at least 1 large group session with Program Chief	• Aids in coordinating, communicates schedules with students, attends all large group sessions, and facilitates problem-based learning cases • Coleads at least 1 large group session with Student Director
Program evaluation	• Reviews program feedback and creates yearly improvement plan with other leaders	• Reviews program feedback and creates yearly improvement plan with other leadership	• Reviews program feedback and creates yearly improvement plan with other leadership

Abbreviation: MAPS = Minority Association of Premedical Students.

### Program Evaluation

Program success and effect were assessed through activity tracking and student evaluation responses. Activity-tracking data included the number of large group meetings attended, in-person meetings with junior and senior mentors, shadowing experiences, and email communications. These data were collected from students who participated in the 2-year pilot. As not all students returned their tracking documentation, the results were extrapolated by creating averages for each category based on the number of students who tracked their activity and then estimated for all students in the program.

Program evaluation responses were collected via anonymous Google Forms survey from program participants. Survey questions varied in type, including 5-point Likert scales, yes/no, and open-ended questions. Students rated the quality of the program, helpfulness of junior and senior mentors, helpfulness of shadowing experiences, ease of navigating shadowing experiences, and helpfulness of large group meetings. Descriptive statistics were used to analyze and report the evaluation data. In addition, students reported any additional benefits from the program besides the stated core components, strengths of the program, and areas of improvement.

### Data Availability

Anonymized data not published within this article will be made available by request from any qualified investigator.

### Funding and Program Support

The program director received initial support from a local grant through the University of Rochester in the form of an inclusive climate leadership fellowship. Subsequently, the program was maintained with minimal funding secured from the University of Rochester Neurology Department's diversity, equity, and inclusion budget. These funds provided stipends for the 2 student leadership positions ($2,000 each per year), lunches during meetings, and transportation to meetings when requested. Once the program was established, the annual funding requirement has been $6,000 per year. The program director and mentor positions have remained volunteer. The program has not required support for an additional administrative individual because the student leaders assumed most of the administrative responsibilities. The PreDoc program students were not specifically compensated, but the program was designed to run during the academic year with contact hours that minimally interfered with their coursework and did not require significant travel.

## Results and Assessment

From 2018 to 2022, the program recruited 29 student participants who completed at least 1 year of the program (27 undergraduate and 2 high school students), including 13 Black or African-American students, 15 Latinx or Hispanic students, and 1 Asian-American student. Eighteen senior (faculty) mentors and 23 junior (medical student) mentors were recruited to participate. Ninety-six percent of undergraduate students completed the full program as of Spring 2022, 2 of the 5 high school students completed 1 year of the program, and 3 of 5 high school students did not adequately engage in the program (Figure). Since the program was run through and funded by the Department of Neurology, most of the senior mentors were neurologists (91% during the pilot, 61% for all program years).

**Figure:**
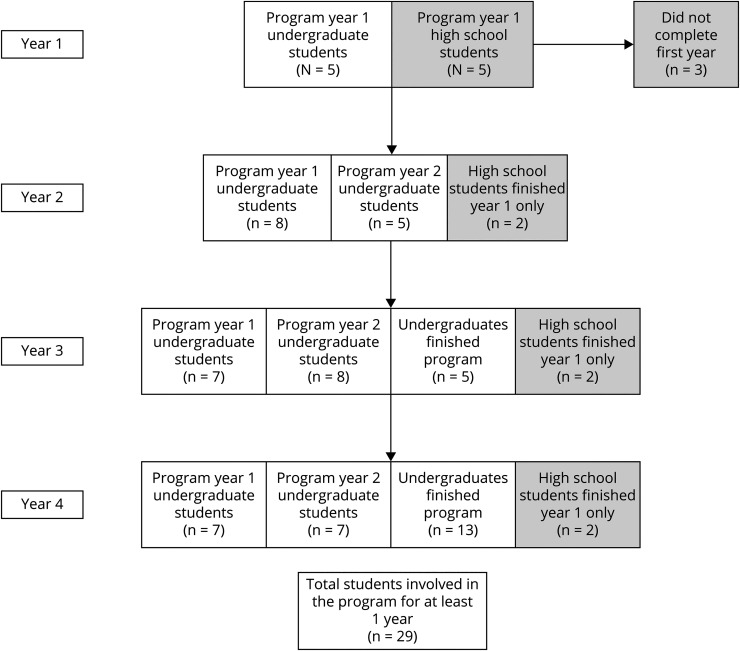
Program Participation Flow Diagram

### Activity Tracking

A total of 15 students from the pilot program (2018–2020) completed at least 1 year of the program (7 students who completed the first year, 5 students who returned for the second year, and 8 students recruited during the second year of the pilot). Accounting for the 5 returning students, there were 20 student-years of data collected for activity tracking. We received a total of 12 returned activity trackers for the 20 student-years (60%), as not every student returned their activity-tracking data. The students met with their senior mentors in-person a median of 2 times per year (range 1–4) and met with their junior mentors in-person a median 1 time per year (range 0–5). They participated in a median 1.5 shadowing experiences (range 0–8), 3 large group meetings (range 2–4), and had 10 email communications with mentors per year (range 5–68). Using the information received and extrapolating for all 20 student-years, we estimated that the program facilitated the following activities during the 2 pilot years: 45 in-person senior mentor meetings, 27 in-person junior mentor meetings, 42 shadowing experiences, 60 large group meetings, and 360 email communications.

### Program Evaluation

A total of 35 anonymous program evaluation surveys were collected from all 4 years of the program, including the pilot, with results reported in Table 4. Students reported that strengths of the program included mentorship relationships (both support and encouragement from, and normalization of physicians), exposure to medicine, the problem-based learning cases and the large group meetings, and connection to additional resources/opportunities. Sixty percent of the students reported benefits from the program besides the stated core components including receiving letters of recommendation for Summer experiences and medical school, and connections made to help facilitate research opportunities. Areas for improvement cited by students included strength of the junior mentor relationships, desire for increased large group meetings, and desire for increased opportunities for socialization outside of the formal meetings. Table 5 shares exemplar quotations from student evaluation responses discussing strengths and areas for improvement.

**Table 4:** Program Evaluation Data Collected From PreDoc Participants Over 4 Academic Years

**Survey question**	**2018–2020 (N = 14)**	**2020–2022 (N = 21)**	**Total (N = 35)**
	Score, mean (median, range)		
Overall quality of the PreDoc program^a^	4.5 (5, 2)	4.8 (5, 1)	4.7 (5, 2)
Helpfulness of senior mentor as a resource^b^	4.6 (5, 2)	4.7 (5, 2)	4.6 (5, 2)
Helpfulness of junior mentor as a resource^b^	3.8 (4, 4)	4.2 (5, 4)	4.1 (4, 4)
Helpfulness and interest level of shadowing experience^b^	4.7 (5, 1)	4.6 (5, 2)	4.7 (5, 2)
Ease around navigating the shadowing experience^c^	4.4 (5, 4)	4.5 (5, 3)	4.5 (5, 4)
Helpfulness of the large group sessions^b^	4.8 (5, 1)	4.7 (5, 2)	4.7 (5, 2)
	% Students replying “yes”		
Students were able to shadow 2 or more times over the academic year	57	71	66
Students reporting receiving additional benefits from the program (besides the stated core components)	64	57	60

a Scored from 1 to 5, with highest score indicating excellent and lowest score indicating poor.

b Scored from 1 to 5, with highest score indicating very helpful and lowest score indicating not at all helpful.

c Scored from 1 to 5, with highest score indicating very easy and lowest score indicating difficult.

**Table 5:** Student Narrative Evaluation Responses Reflecting on Their Experience With the Program

**Strength**	**Narrative responses**
Support from physicians	“Just knowing that we have extra support and people that encourage us is really helpful. I have no doctors in my family and sometimes I get lost because I don't know what step to take or just have someone to watch over my step to make sure I'm taking advantage of any opportunities I can. That is a big one for me.” “[My mentor] helped me with more of my mental awareness issues. This is something people wouldn't expect to come out from the program. However, the college journey has been pretty difficult for me so it was nice and helpful to have someone who has been through it really believe in me.”
Normalization of physicians	“…mentors were a constant part of our lives so the shadowing didn't stop when we left the clinic or Zoom. For [me] or anyone who does not have any physicians in their families, doctors seem kind of foreign, they're just people you see once in a while. For me it was really cool to kind of normalize physicians, learn about their experiences and see who they are when they're not clocked in.”
Exposure to clinical medicine	“I found the overall exposure to medicine extremely helpful. I've been able to make more connections (speakers, my mentor, the other medical students) and gain so much information about different specialties, applying to med school, etc. It's made the whole pre-med experience seem less daunting.”
Connection with resources and opportunities	“My mentor will be writing a letter of recommendation to supplement my medical school application. Also, she and I received funding through the AAN to attend their conference and present our work there.”
Areas for improvements	
Importance of near peer mentoring	“If possible, maybe try to find junior mentors that are in their first or second year. Mine was great but she was a fourth year so it was a bit harder to relate to her and she was very busy doing her rotations. She also had forgotten a little bit about the med school application process… Also, I would say to try to get the mentees to get to know each other better…”
Increase exposure to practical skills	“I feel we could do more application-based things, maybe like mock interviews, personal statement help, and how to effectively narrow down a list of medical schools.”

Abbreviation: AAN = American Academy of Neurology.

## Discussion and Lessons Learned

We successfully created a longitudinal, clinically focused pipeline program requiring minimal funding for undergraduate students who identified with a racial or ethnic background historically minoritized in medicine. The program was directed by an academic neurologist and primarily run through the Department of Neurology, thus providing students with increased exposure to the field, including a total of 507 interactions over the pilot 2 years with majority neurologists. Students found their senior mentors, shadowing experiences, and large group meetings especially helpful. These aspects of the program offered mentorship and increased knowledge of the field, both of which are cited factors that retain students on the premedical education track.^13^ Students were also exposed to the general roles and responsibilities of an academic neurologist including scholarly work, teaching, and conferences. Our activity-tracking data support the feasibility and success in building these longitudinal relationships between students and their mentors.

Our program evaluation indicated that students were very satisfied with the overall quality of the program (Table 4). We feel that success was likely improved and sustained by the addition of the student leaders who helped add programming most relevant to our students and worked to support the mentor-mentee relationships. Helpfulness of the senior mentor was also a clear highlight as evidenced by the high rating. Evaluations commented on support (e.g., mental support and letters of recommendations), normalization of physicians, exposure to the field, and connections made. The helpfulness of shadowing experiences and perceived ease of shadowing were rated highly. Large group sessions were also popular with the students because they allowed students to come together as a group, provided professional development learning, and got students excited to think through medical cases similar to what they would experience in medical school.

Leadership of the program was eventually split into 3 different positions, which helped to minimize the time requirement for the director and provide leadership opportunities for students. Although many medical centers are prioritizing diversity, equity, and inclusion, the financial support of pipeline programs is often limited. After initial salary support for the director to create the program, the director position is now volunteer, and only minimal support is required for student leader stipends, large group meeting lunches, and student transportation costs (currently covered by the Department of Neurology). Faculty and medical student volunteer mentors can expect their experiences to be rewarding and lead to personal and professional development, and may qualify faculty for incentive salary support in some departments.^20^ In addition, participation in the program may allow neurology faculty to meaningfully broaden their perspectives and experiences with individuals of differing backgrounds. This is particularly important for faculty who do not themselves identify as minoritized, and it is important for such faculty to be involved in these initiatives to reduce the “diversity tax” experienced by our racial and ethnically minoritized faculty. Although our program was maintained with minimal funding and no compensation for mentors, we hope that Neurology Departments will increasingly recognize the importance and create funding opportunities for mentors involved in this work.

During the first 3 years of the program, all undergraduate students were recruited directly from the University of Rochester. As such, those students had already experienced some degree of privilege relative to other members of the same racial and ethnic group. We felt it was important to consider the relative privilege that some racial and ethnically minoritized individuals may have over others and thus took into account during student selection, factors that could indicate less access to role-models within medicine, to medical experiences, and to high quality prior education. However, we needed to balance those considerations with admitting students who were likely to succeed in the program. After the first 3 years, we attempted to further address this by opening up the program to other area colleges.

We have discovered some areas for improvement and have worked through yearly improvement plans. After the first year, it became apparent the program was better equipped for undergraduate rather than high school students, so during all subsequent years, we recruited only undergraduate students. In our experience with the few high school students admitted into the program, it was more difficult for them to attend the large group meetings during the school year (perhaps due to transportation challenges and/or high school–related activities). They also tended to be less communicative with their mentors. In addition, our institution requires that students be 16 years or older for shadowing. This created a small window for students who were old enough to shadow, but young enough to spend 2 years in the program before graduating high school. The 2 students who completed 1 year of the program were both reasonably engaged but were in 12th grade and left for college before the second year.

Helpfulness of junior mentors was one of the lowest scored aspects of the program. Initial student evaluation data revealed that some students had difficulty connecting with their junior mentor or wanted to meet with their junior mentor more often and desired more information about the medical school application process. We found that students in the program were often hesitant to reach out to their junior mentors, which put the onus of the relationship on the mentor. One student felt the junior mentors should be early in their medical education, so they would have experienced the medical school application process most recently. It is possible that some of the junior mentors did not view their role as a priority and, as a result, failed to meet the expectations outlined by the program.^21^ To address these concerns, we established the leadership role of Student Director (Table 3), an individual who helps organize and track the junior mentor activities. We also created a checklist for the junior mentors to help them stay on track with the expected activities.

The percentage of students who were able to shadow 2 or more times during the academic year was lowest during the 2019–2020 academic year likely due to the COVID-19 pandemic. The early pandemic contributed to our students only shadowing a median of 1.5 times per year in our pilot, despite our goal of 2–3 experiences per year. For the safety of learners and patients, most hospitals limited observational learning opportunities.^22^ However, despite the pandemic persisting into the 2020–2021 year, the percentage of students who were able to shadow at least twice in that academic year increased, as opportunities to virtually shadow increased, without decreasing the helpfulness of the shadowing experiences. We found that virtual shadowing worked quite well in exposing students to health care in action. It was particularly useful to allow for brief intervals between patients where the student and preceptor stay on video to debrief the previous patient and introduce the next patient. It was also useful to prompt the students beforehand on items such as proper dress, professional background, and an understanding of how the clinic would run. The main challenge to virtual formatting involved the large group meetings, which were all held virtually during the 2020–2021 academic year. It was harder to engage students on video, and much more challenging to create a sense of community and connection among the students.

Throughout the program, we provide students with exposure to individuals with concordant racial/ethnic identities through various means, such as speakers at large group meetings and junior mentors. Moving forward, we hope to increase the number of senior mentors who themselves identify as minoritized because students benefit from having role models in medicine within whom they can see themselves.^23^ Yet, we will continue engaging faculty who do not identify as minoritized so as to reduce the “diversity tax” experienced by our racial and ethnically minoritized faculty. Overall, we feel that it is important to engage all groups in this work. However, it is important to have robust mentor training strategies to ensure that all individuals are competent to mentor this population of students.

There were some identified limitations to our evaluation of the program. We were not able to receive evaluations or activity tracker data from all program participants, so we needed to extrapolate the data for the whole group. It is possible that the experiences of the students who completed and returned their activity trackers were different from those who did not. A long-term goal is to assess outcomes of the program by tracking student career trajectory and the percentage of students who matriculate into a school for healthcare providers. However, we will not be able to adequately assess those data until more students have completed their undergraduate education and any gap years.

A recent publication elucidated the importance of “creating systematic ways to facilitate interaction with neurologists through both communication opportunities and mentoring relationships… especially for those who identify as underrepresented minorities to strengthen the neurology pipeline and increase diversity.”^15^ We believe that this program is a readily reproducible, low expense, and effective way of doing just that. The overall reception of the program by students and mentors has been overwhelmingly positive, and the program has been found to be sustainable with minimal support after 4 academic years. As the program is clinically oriented and runs during the academic year, it serves as an effective compliment to the many excellent summer research opportunities. We will work to track graduates of the program to understand its effectiveness. Ultimately, we hope our program serves as a template for other Neurology Departments to create pipeline programs that require minimal support and help engage faculty in diversity initiatives and professional development. Ultimately, we hope the program will inspire more students who identify from a racial or ethnic background historically marginalized in Neurology to succeed in medicine, specialize in neurology, and increase representation within the field to the benefit of our patients, our trainees, and ourselves.

## Appendix Authors

**Table:**

**Name**	**Location**	**Contribution**
Shane Fuentes, BS	University of Rochester, NY	Drafting/revision of the manuscript for content, including medical writing for content; study concept or design; analysis or interpretation of data
Rachel M.E. Salas, MD, MEd	Johns Hopkins University, Baltimore, MD	Drafting/revision of the manuscript for content, including medical writing for content; study concept or design
Olivia Brumfield, BS	University of Rochester, NY	Drafting/revision of the manuscript for content, including medical writing for content; study concept or design
Robert Thompson Stone, MD	University of Rochester, NY	Drafting/revision of the manuscript for content, including medical writing for content; major role in the acquisition of data; study concept or design; analysis or interpretation of data

## Glossary

COVID-19: coronavirus disease 2019
GPA: grade point average
JHM: Johns Hopkins Medicine
STEP: Science and Technology Entry Program
